# Influence of androgenic blockade with flutamide on pain behaviour and expression of the genes that encode the NaV1.7 and NaV1.8 voltage-dependent sodium channels in a rat model of postoperative pain

**DOI:** 10.1186/s12967-019-2031-z

**Published:** 2019-08-27

**Authors:** José Osvaldo Barbosa Neto, João Batista Santos Garcia, Maria do Socorro de Souza Cartágenes, Andressa Godoy Amaral, Luiz Fernando Onuchic, Hazem Adel Ashmawi

**Affiliations:** 1 LIM/08 – Laboratório de Anestesiologia – Laboratórios de Investigação Médica do Hospital das Clínicas da Faculdade de Medicina da Universidade de São Paulo, São Paulo, Brazil; 2 Experimental Laboratory to the Study of Pain – Universidade Federal do Maranhão, São Luís, Brazil; 3 LIM/29 – Laboratório de Nefrologia Celular, Genética e Molecular – Laboratórios de Investigação Médica do Hospital das Clínicas da Faculdade de Medicina da Universidade de São Paulo, São Paulo, Brazil

**Keywords:** Postoperative pain, Gender influence, Testosterone, NAV1.7 and NAV1.8 voltage-gated sodium channels, *Scn9a* e *Scn10a* gene expression

## Abstract

**Background:**

Experimental studies suggest that testosterone reduces the nociceptive response after inflammatory and neuropathic stimuli, however the underlying mechanisms have not been fully elucidated. The aims of this study were to evaluate the effect of peripheral blockade of testosterone on pain behaviour and on expression levels of the genes that encode the NaV1.7 and NaV1.8 channels, in dorsal root ganglia in an acute postoperative pain model, as well as the influence of androgen blockade on the expression of these genes.

**Methods:**

Postoperative pain was induced by a plantar incision and the study group received flutamide to block testosterone receptor. The animals were submitted to behavioural evaluation preoperatively, 2 h after incision, and on the 1st, 2nd, 3rd and 7th postoperative days. Von Frey test was used to evaluate paw withdrawal threshold after mechanical stimuli and the guarding pain test to assess spontaneous pain. The expression of the genes encoding the sodium channels at the dorsal root ganglia was determined by real time quantitative polymerase chain reaction.

**Results:**

Animals treated with flutamide presented lower paw withdrawal threshold at the 1st, 2nd, 3rd, and 7th postoperative days. The guarding pain test showed significant decrease in the flutamide group at 2 h and on the 3rd and 7th postoperative days. No difference was detected between the study and control groups for the gene expression.

**Conclusions:**

Our data suggest an antinociceptive effect of androgens following plantar incision. The expression of genes that encode voltage-gated sodium channels was not influenced by androgen blockade.

## Background

The phenomenon of pain receives the influence of several systems in the body, including the limbic, adrenergic, endogenous opioid and hormonal systems. Notably, the latter has been receiving more attention in the last 20 years. Some studies have focused on the potential relationships between gonadal hormones and pain behaviour, having revealed that it may be intensified by female gonadal hormones [1–5]. Data on male hormones, however, are scarce. The observation that patients with temporomandibular chronic pain and low testosterone levels display higher pain expression indicates that this hormone may modulate pain [6, 7]. In male rats, testosterone presented an antinociceptive effect in models of inflammatory pain (carrageenan, formalin and Freud’s adjuvant) [7–10]. The mechanisms underlying such effects of sex hormones, however, are not fully understood.

Gonadal hormones appear to increase expression of voltage-dependent sodium channels (NaV channels) in the trigeminal ganglion after inflammatory stimulation with formalin [1, 11]. NaV channels are crucial in neuronal excitation and signalling and are expressed at various sites of the nervous system, including primary afferents and the dorsal root ganglion (DRG).

Studies have demonstrated increased expression of NaV1.7 channels after inflammation [1, 11], an increase that contributes to exacerbation of pain behaviour [12–14]. Likewise, the role of NaV1.8 was established in inflammatory pain, where knockout animals for its encoding gene had reduced response to thermal and chemical stimuli [15, 16]. In studies that assessed the influence of estradiol on the modulation of such channel’s expression, increased NaV1.7 expression was observed. Whether testosterone has the same potential to influence their expression, however, remains unknown [1].

To expand the knowledge on the relationship between pain and testosterone, the current study evaluated the effect of peripheral blockade of testosterone on pain behavior and quantified Scn9a and Scn10a expression (rat orthologs of human SCN9A and SCN10A, the genes that encode NaV1.7 and NaV1.8 sodium channels, respectively) in DRG using a rat model of acute postoperative pain.

## Method

The study was divided in a behavioural evaluation to assess differences in responses to painful stimuli and real time reverse transcription polymerase chain reaction (RT-PCR) assays to evaluate the expression levels of *Scn9a* (NaV1.7) and *Scn10a* (NaV1.8). The rats were randomly assigned to Flutamide (F) and Control (C) groups.

**Fig. 1:**
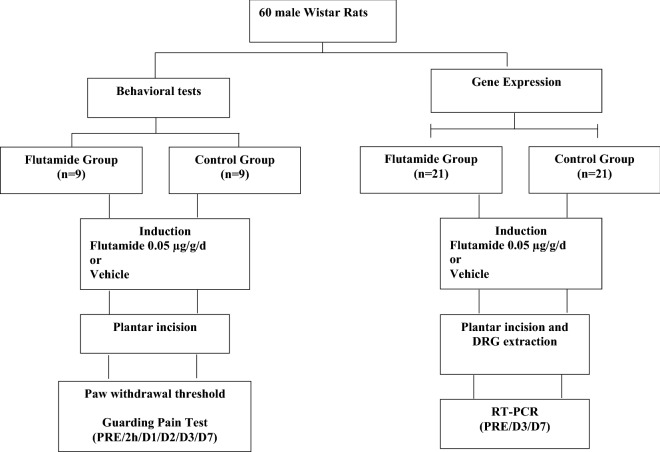
Study flowchart

Prior to each stage of the study, the animals were exposed to flutamide preparation administered by a blinded researcher other than the one who performed the behavioural tests. Group F rats received flutamide (Sigma-Aldrich, Brazil) at a dosage of 0.050 µg/g of animal weight/day, injected into the animals’ dorsal region subcutaneously for the 7 days prior to the experiment. The drug was diluted in a solution of propylene glycol/10% ethanol because the solubility of this vehicle is higher than that of vegetable oils. Flutamide is a non-hormonal medication that produces an anti-androgenic effect through its binding to androgen receptors, preventing the coupling of androgens to their receptors, and reduces the uptake of testosterone by peripheral tissues (Affaitati et al. [27]). Group C animals received only the propylene glycol solution/10% ethanol by the same administration route. After the preparation phase, the animals were submitted to the plantar incision pain model of acute postoperative pain for behavioural and gene expression experiments. The researcher in charge of this investigation was blinded to the animal group allocation (Fig. 1).

The behavioural tests were performed at the following times: Pre-incision (PRE); 2 h after incision (2 h); and on the 1st (D1), 2nd (D2), 3rd (D3) and 7th (D7) postoperative days. Gene expression was studied at PRE, D3 and D7.

### Sample size

Sample size was calculated using G*Power software [17]. For the behavioral study, the number of animals was calculated using t-student test to obtain a 30% difference between groups for the paw withdrawal threshold test, with 80% power and 0.05 alfa error, obtaining 9 animals per group.

For the gene expression experiments, DRG samples were obtained from seven animals for each time point and for each group (total = 42). Sample size was calculated using ANOVA test with 80% power and 0.05 alfa error, obtaining 7 animals per group.

### Plantar incision postoperative acute pain model

The animals were submitted to plantar incision of the right hind paw under general anaesthesia with 2–3% isoflurane. The procedure was performed aseptically as described by Brennan et al. [18].

### Behavioral tests

Paw withdrawal threshold test [18]: The rats were placed in individual boxes with enough space to move around and kept on a metal screen with openings of 1 cm^2^. After a period of adaptation to the site, paw withdrawal threshold measurements were performed after mechanical stimulation with von Frey’s digital algesimeter (Insight Ltda, Ribeirão Preto, SP, Brazil). The mechanical stimulus was limited to 60 g to prevent worsening of the lesion and was applied to the right hind paw plantar region, close to the incision scar. Five measurements were obtained at intervals of 2 min (min) and the mean calculated, representing the test final value.

Guarding Pain Test [18]: A cumulative score was used to assess non-evoked pain behaviour. It was always performed prior to the evoked pain test to avoid confounders. Similarly, to the previous test, the animals were placed on a metal screen with openings of 1 cm^2^.

Incised and non-incised hind paws were carefully examined during a period of 1 min. This procedure was repeated every 5 min for 1 h (h). During the evaluation, scores ranging from 0 to 2 were assigned depending on the positioning most often adopted by each paw of the animal during the observation period.

The scores were assigned as follows: 0 = The surgical wound of the animal’s paw is totally in contact with or even distorted by the net or appears whitish. 1 = The rat paw gently touches the net, but without distortion of the surgical wound or a whitish appearance. 2 = The paw does not contact the net.

The sum of the 12 scores (0–24) obtained during 1 h of observation was recorded. The difference between the scores obtained for the healthy and incised paws represents the cumulative pain score.

At the end of the experiment, the animals were euthanized via cardiac puncture exsanguination under general anaesthesia with isoflurane.

### Real-time quantitative polymerase chain reaction (RT-qPCR)

RT-qPCR was used to evaluate mRNA expression to determine whether surgical trauma-related hyperalgesia derives from change in expression of voltage-dependent sodium channels in the DRG, and whether this expression can be modulated by blocking testosterone receptors.

To obtain the DRG samples, the animals were anaesthetized according to protocol. To reduce bleeding in the operative field and thus the time to obtain the samples, the rats were submitted to thoracotomy and sacrificed similarly to the previously described method. Laminectomy was then performed to obtain the dorsal root ganglia of L4 and L5 ipsilateral to the plantar incision. The samples were frozen in liquid nitrogen and stored at − 80 °C.

Total RNA was extracted using the ReliaPrep™ RNA Miniprep Systems Kit (Promega, Madison, WI) according to the manufacturer’s instructions. The obtained RNA was subjected to spectrophotometric quantification with Nanodrop 1000 (Thermo Fisher Scientific, Waltham, MA) and only samples with an A260/A280 ratio higher than 1.8 were used. RNA integrity was analyzed by automated electrophoresis in the Agilent 2100 TapeStation Bioanalyzer (Agilent Technologies, Santa Clara, CA). Samples with RIN above 7.2 were included in this study. Synthesis of cDNA by reverse transcriptase was carried out using the IMProm-II Reverse Transcription System (Promega, Madison, WI). Gene expression analysis was performed using *TaqMan* specific assays for *Scn9a* (Rn00591020_ml), *Scn10A* (Rn00568398_ml) and TATA box protein/TBP (Rn01455646_ml, Thermo Fisher Scientific, Waltham, MA) as control. Results were obtained with the Ct methodology and expressed as arbitrary units.

### Statistical analysis

All behavioural analyses were performed using Prism 4.0 software (GraphPad Software, Inc., USA). Data normality was assessed using the Kolmogorov–Smirnov test. The graphs present the data presented as median, lower and upper quartile. The variance in the results obtained in the behavioural tests and in gene expression analyses as a function of time was evaluated by the Kruskal–Wallis test, while the differences between the groups were determined by the Mann–Whitney test for each time point. A p value < 0.05 was considered statistically significant.

## Results

### Evoked pain (paw withdrawal threshold)

**Fig. 2:**
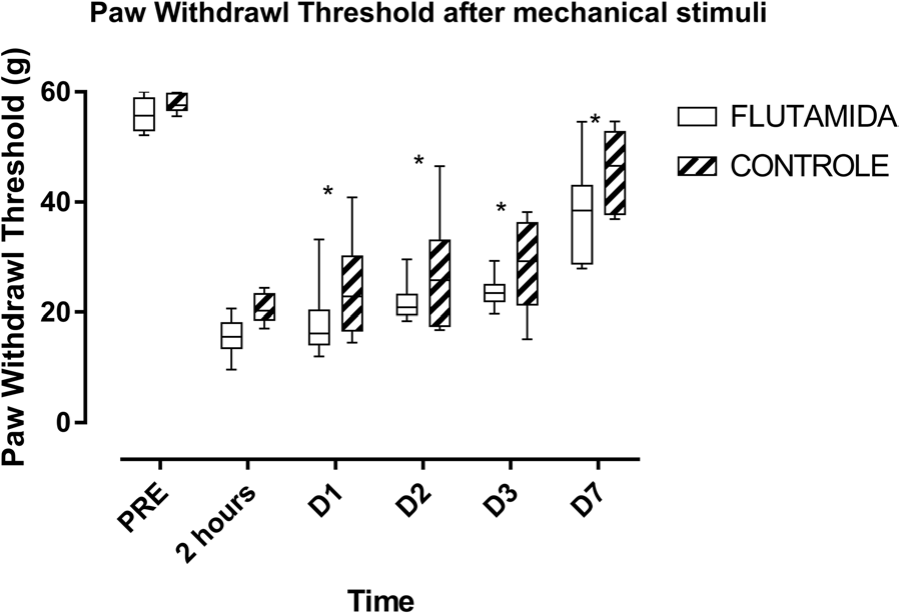
Pattern of the paw withdrawal threshold in Flutamide-treated and Control rats. The data were analyzed using the Mann–Whitney test and are presented as median, lower and upper quartile. *p < 0.05 versus Control group

After implementation of the postoperative pain model by plantar incision, both groups developed mechanical hyperalgesia and exhibited a reduced paw withdrawal threshold, which reached its lowest analyzed value at 2 h (median values: 15.2 vs 24.1 for the Flutamide and the Control groups, respectively). The pain threshold for paw withdrawal was significantly lower than PRE at 2 h and on D1, D2, D3 and D7 for both groups (Fig. 2).

All animals progressively recovered from hyperalgesia; however, they did not recover their pre-incision status until the seventh postoperative day. Of note, rats treated with flutamide developed lower pain threshold for paw withdrawal than control animals on D1, D2, D3 and D7 (Fig. 2).

### Evaluation of spontaneous pain (Guarding Pain Test)

**Fig. 3:**
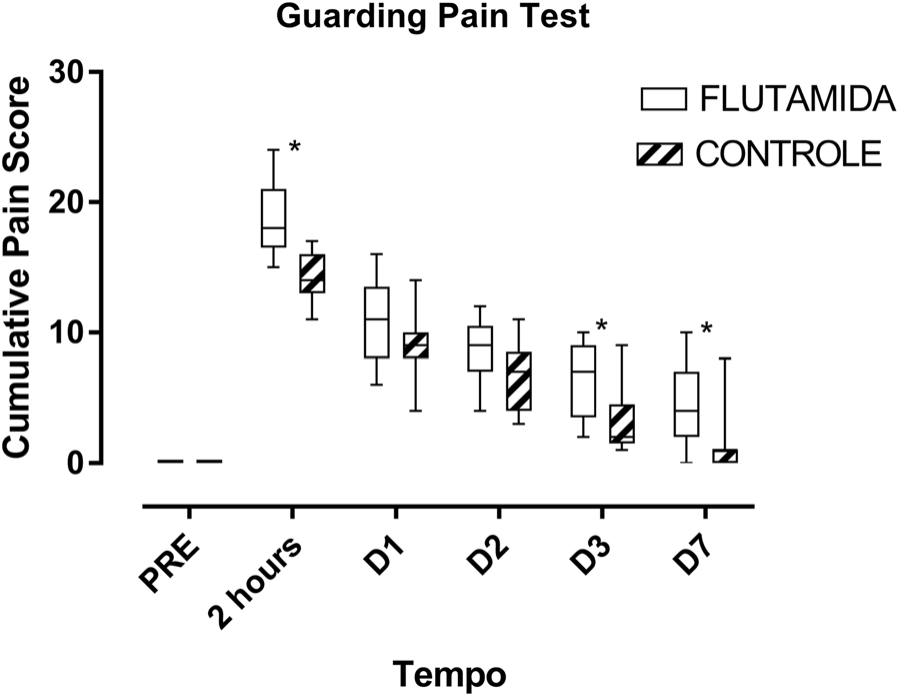
Spontaneous pain represented by Guarding Pain test scores over time for the flutamide-treated and non-treated groups. Data analyzed with the Mann–Whitney test and expressed as median, lower and upper quartile. *p < 0.05 versus Control group

The animals avoided touching the bars of the cage floor more frequently after plantar incision in both groups, and this behaviour was most evident in the assessment performed 2 h after surgery. The Guarding Pain Test median scores reached 18 and 14 for the rats treated with flutamide and the control rats, respectively. Analysis of variance showed that such scores were influenced by treatment with flutamide (H = 88.9 and p < 0.0001). Treated rats displayed significantly higher scores than non-treated animals throughout the evaluation period at 2 h, D3 and D7, with trends in the same direction on D1 and D2 (Fig. 3).

As in the evoked pain test, animal behaviour gradually returned to preoperative levels, reaching its lowest scores on D7 (4 vs 1 for the Flutamide and Control groups, respectively) (Fig. 3).

### Rt-pcr

**Fig. 4:**
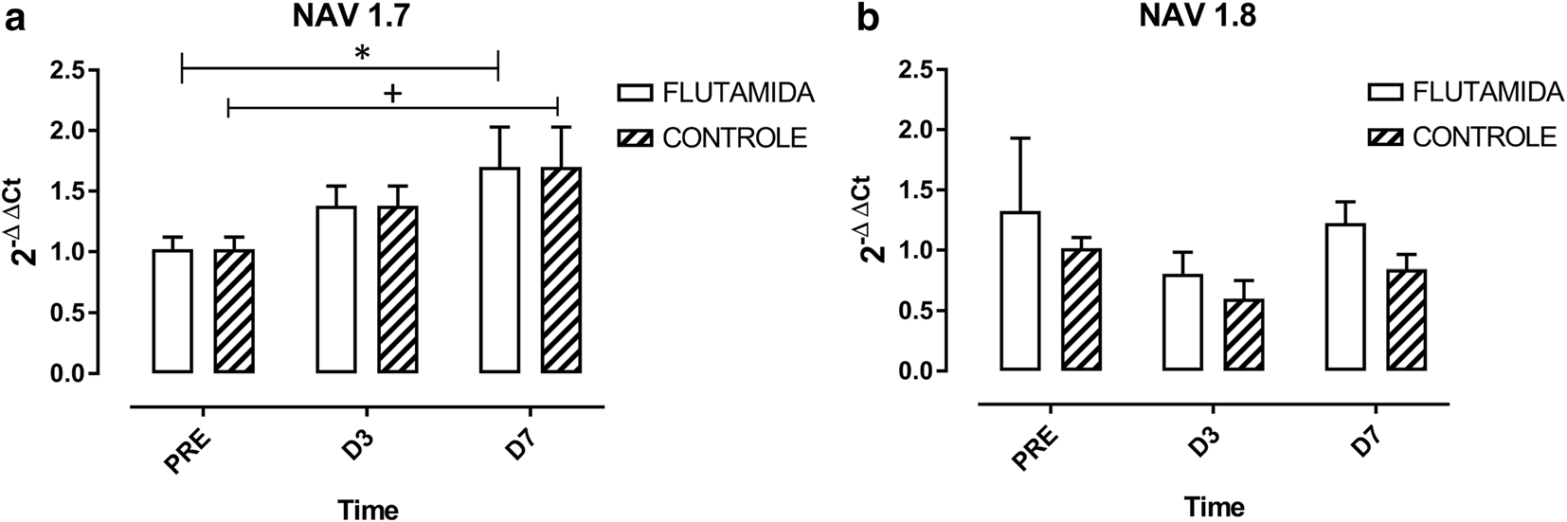
*Scn9a* (**a**) and *Scn10a* (**b**) expression by real-time qPCR in DRG of flutamide-treated and non-treated rats. N = pool of DRG mRNA from seven animals per group for each time point. Data analyzed with the Mann–Whitney test and expressed as median, lower and upper quartile. *p < 0.05. Variance analysis (Kruskal–Wallis test) showed that NaV1.7 expression was dependent on time in both groups. *p < 0.05 versus pre-incision; +p<0.05 versus pre-incision

Animals of both groups showed increased expression of *Scn9a* (NaV1.7) in DRG on D7 compared to pre-incision values (H = 13.11, p = 0.0224) (Fig. 4). *Scn10a* (NaV1.8) expression, however, did not significantly change following incision, on D3 and D7 (H = 6.578, p = 0.254).

## Discussion

Our data revealed that androgenic blockade promoted exacerbation of nociception in a classical rat pain model, supporting the concept that androgens are associated with antinociception. This conclusion was based on a lower threshold for paw withdrawal and more evident guarding behaviour of the incised hind paw developed by flutamide-treated animals. Interestingly, gene expression analyses showed increased *Scn9a* expression in DRG post-incision whereas *Scn10a* expression did not significantly vary after this trauma. Flutamide did not modulate, however, the expression of *Scn9a*.

The plantar incision model has been used to better reproduce the mechanisms observed in acute postoperative pain, facilitating the translation of results to humans and to clinical practice [18]. Plantar incision causes a state of hyperalgesia by inducing the production of local inflammatory cytokines, neural growth factor (NGF) [19] and brain-derived growth factor in DRG [20], in addition to an increase in the spontaneous activity of DRG neurons [18].

The scores based on the guarding pain test were similar in the two groups, peaking at 2 h and then decreasing progressively over time. The same trend was observed for the paw withdrawal threshold, which reached the lowest value at 2 h and gradually increased until the seventh postoperative day in both groups. This pattern is similar to the ones reported in other studies using the same experimental model, where the peak of hyperalgesia occurred in the first postoperative hours and was followed by spontaneous resolution around the tenth postoperative day [18, 19, 21].

The animals treated with flutamide presented a lower paw withdrawal threshold compared to the controls, as well as higher scores in the spontaneous pain test. The results associated with the behavioural tests suggest a greater degree of hyperalgesia in animals submitted to testosterone blockade by flutamide. The antinociceptive action of testosterone has already been demonstrated in acute inflammatory pain. Orchiectomized animals submitted to carrageenan injection in the paw presented more pronounced exacerbation of hyperalgesia than controls, whereas testosterone replacement reversed this difference [22]. Additionally, after formalin injection orchiectomized males exhibited potentiated pain behaviour, while females receiving testosterone exhibited less pain [2, 23]. The plantar incision model was also used to compare postoperative pain between males and females, but no difference was observed in pain behaviour or response to opioid analgesics [24].

Although several studies have used surgical castration as means of inducing hypogonadism, this method has the disadvantage of impairing the control of physiological mechanisms related to sex hormones, leading to inadequate maintenance of elevated or depleted hormonal levels [25]. Wistar and Sprague–Dawley rats presented high plasma oestradiol concentrations as a result of orchiectomy, reaching levels equivalent to those found in females of these strains [26]. Flutamide is used to achieve androgenic blockade without the complications of surgical castration [27]. A recent study demonstrated that the analgesic effect of testosterone is dependent on activation of the testosterone receptor and that flutamide reversed this protective effect after formalin injection into the temporomandibular joint (TMJ) [28].

The evidence accumulated over the last decade corroborates the results obtained in the present study, suggesting a protective role of testosterone against pain, but the underlying mechanisms are still unclear [8, 22, 23, 28, 29]. The increase in the expression of voltage-dependent sodium channels, particularly NaV1.7 and NaV1.8, has already been demonstrated in previous studies using inflammatory pain models [15, 30, 31]. It was suggested that oestradiol might modulate the expression of these channels. This hypothesis was in fact confirmed by a study where animals were treated with the oestrogen receptor blocker ICI 182,780. In this case, NaV1.7 expression was increased in the trigeminal ganglion in a model of inflammation of the TMJ, with concomitant increase in mechanical hyperalgesia [1, 11].

*Scn9a* expression increased in the present study following the established surgical trauma, a pattern that did not occur for *Scn10a*. This lack of increase in NaV1.8 expression corroborates data reported by previous studies evaluating the role of sodium channels in postoperative pain [12, 15]. Despite the increase in *Scn9a* expression, it was apparently not related to testosterone effects, therefore not participating in mechanisms of testosterone-induced analgesia. Our data thus support that androgenic blockade increases pain behaviour in rats, suggesting an antinociceptive role for testosterone, not related to modulation of NaV1.7 channel expression.

The choice of using L4 and L5 DRGs was based on the fact that the hindpaw innervation of these dermatomes are made by these nerve roots. We believe that if L3 or L6 DRGs had been added, it would have been more difficult to obtain a more precise change in NaV channel expression. One possibility that was not explored in this paper is the study of dorsal horns along with the DRGs, that might bring relevant additional information to the field.

Recently, a possible role of the central opioid system in the protective effect of testosterone was described. After treatment with naloxone, gonadectomized male rats on testosterone replacement that received formalin TMJ injection presented increased behavioral response, while the opioid antagonist had no effect on gonadectomized animals without hormonal replacement [7]. Additional evidence of a relationship between androgens and opioid system supports this hypothesis. An androgen receptor (AR) binding site was found in the promoter region of the mu opioid receptor (MOR) gene Oprm1. In the presence of testosterone, AR can transcriptionally activate Oprm1 and amplify the opioid receptor synthesis. This effect was not observed after AR blockade [32].

Some limitations may be considered in our study. The use of subcutaneous route facilitates the injection of the study drug; however, it carries the risk of erratic absorption. Moreover, the chosen time points to evaluate the expression levels of *Scn9a* and *Scn10a* may not have been sufficient to obtain accurate time expression curves. Also, the stimuli provided by the plantar incision may not have been intense or long enough to induce amplification of genes that encode NaV channels.

## Data Availability

The datasets used and/or analysed during the current study are available from the corresponding author on reasonable request.

## Abbreviations

AR: androgen receptor
DRG: dorsal root ganglion
H: hours
Min: minutes
NaV: voltage-dependent sodium
NGF: neural growth factor
OPRM1: mu opioid receptor gene
PRE: pre-incision
RT-PCR: real time reverse transcription polymerase chain reaction
RT-qPCR: real-time quantitative polymerase chain reaction
TMJ: temporomandibular joint

## References

[1] Bi RYY, Meng Z, Zhang P, Wang XDD, Ding Y, Gan YHH (2017). Estradiol upregulates voltage-gated sodium channel 1.7 in trigeminal ganglion contributing to hyperalgesia of inflamed TMJ. PLoS ONE.

[2] Gaumond I, Arsenault P, Marchand S (2002). The role of sex hormones on formalin-induced nociceptive responses. Brain Res.

[3] Yamagata K, Sugimura M, Yoshida M, Sekine S, Kawano A, Oyamaguchi A (2016). Estrogens exacerbate nociceptive pain via up-regulation of trpv1 and ano1 in trigeminal primary neurons of female rats. Endocrinology.

[4] Lu Y-C, Chen C-W, Wang S-Y, Wu F-S (2009). 17Beta-estradiol mediates the sex difference in capsaicin-induced nociception in rats. J Pharmacol Exp Ther.

[5] Li L, Fan X, Warner M, Xu XJ, Gustafsson JÅ, Wiesenfeld-Hallin Z (2009). Ablation of estrogen receptor α or β eliminates sex differences in mechanical pain threshold in normal and inflamed mice. Pain.

[6] Tonsfeldt KJ, Suchland KL, Beeson KA, Lowe JD, Li M-H, Ingram SL (2016). Sex differences in GABA A signaling in the periaqueductal gray induced by persistent inflammation. J Neurosci.

[7] Macedo CG, Fanton LE, Fischer L, Tambeli CH (2016). Coactivation of mu- and kappa-opioid receptors may mediate the protective effect of testosterone on the development of temporomandibular joint nociception in male rats. J Oral Facial Pain Headache.

[8] Fischer L, Clemente JT, Tambeli CH (2007). The protective role of testosterone in the development of temporomandibular joint pain. J Pain.

[9] Flake NM, Hermanstyne TO, Gold MS (2006). Testosterone and estrogen have opposing actions on inflammation-induced plasma extravasation in the rat temporomandibular joint. Am J Physiol Regul Integr Comp Physiol.

[10] Aloisi AM, Affaitati G, Ceccarelli I, Fiorenzani P, Lerza R, Rossi C (2010). Estradiol and testosterone differently affect visceral pain-related behavioural responses in male and female rats. Eur J Pain.

[11] Bi RYY, Ding Y, Gan YHH (2015). A new hypothesis of sex-differences in temporomandibular disorders: estrogen enhances hyperalgesia of inflamed TMJ through modulating voltage-gated sodium channel 1.7 in trigeminal ganglion?. Med Hypotheses.

[12] Li Z, Li Y, Cao J, Han X, Cai W, Zang W (2017). Membrane protein Nav1.7 contributes to the persistent post-surgical pain regulated by p-p65 in dorsal root ganglion (DRG) of SMIR rats model. BMC Anesthesiol.

[13] Cai W, Cao J, Ren X, Qiao L, Chen X, Li M (2016). shRNA mediated knockdown of Nav1.7 in rat dorsal root ganglion attenuates pain following burn injury. BMC Anesthesiol.

[14] Xia Z, Xiao Y, Wu Y, Zhao B (2016). Sodium channel Nav1.7 expression is upregulated in the dorsal root ganglia in a rat model of paclitaxel-induced peripheral neuropathy. Springerplus.

[15] Joshi SK, Mikusa JP, Hernandez G, Baker S, Shieh CC, Neelands T (2006). Involvement of the TTX-resistant sodium channel Nav 1.8 in inflammatory and neuropathic, but not post-operative, pain states. Pain.

[16] Kerr BJ, Souslova V, McMahon SB, Wood JN (2001). A role for the TTX-resistant sodium channel Nav 1.8 in NGF-induced hyperalgesia, but not neuropathic pain. Neuroreport.

[17] Buchner A, Paul F, Erdfelder E, Albert-georg L (2014). G*Power 3.1 (manual): a flexible statistical power analysis program for the social, behavioral, and biomedical sciences. Behav Res Methods.

[18] Brennan TJ (2011). Pathophysiology of postoperative pain. Pain.

[19] Spofford CM, Brennan TJ (2012). Gene expression in skin, muscle, and dorsal root ganglion after plantar incision in the rat. Anesthesiology.

[20] Yuan XH, Liu XY, Tang QP, Deng YL (2013). Pain-related mediators underlie incision-induced mechanical nociception in the dorsal root ganglia. Neural Regen Res.

[21] Wu C, Erickson MA, Xu J, Wild KD, Brennan TJ (2009). Expression profile of nerve growth factor after muscle incision in the rat. Anesthesiology.

[22] Borzan J, Fuchs PN (2006). Organizational and activational effects of testosterone on carrageenan-induced inflammatory pain and morphine analgesia. Neuroscience.

[23] Aloisi AM, Ceccarelli I, Fiorenzani P, De Padova AM, Massafra C (2004). Testosterone affects formalin-induced responses differently in male and female rats. Neurosci Lett.

[24] Kroin JS, Buvanendran A, Nagalla SKS, Tuman KJ (2003). Postoperative pain and analgesic responses are similar in male and female Sprague-Dawley rats. Can J Anesth.

[25] Greenspan JD, Craft RM, LeResche L, Arendt-Nielsen L, Berkley KJ, Fillingim RB (2007). Studying sex and gender differences in pain and analgesia: a consensus report. Pain.

[26] Craft RM, Mogil JS, Maria Aloisi A (2004). Sex differences in pain and analgesia: the role of gonadal hormones. Eur J Pain.

[27] Affaitati G, Ceccarelli I, Fiorenzani P, Rossi C, Pace MC, Passavanti MB (2011). Sex differences in the analgesic effects of ICI 182,780 and Flutamide on ureteral calculosis in rats. Horm Behav.

[28] Fanton LE, Macedo CG, Torres-Chávez KE, Fischer L, Tambeli CH (2017). Activational action of testosterone on androgen receptors protects males preventing temporomandibular joint pain. Pharmacol Biochem Behav.

[29] Torres-Chávez KE, Sanfins JM, Clemente-Napimoga JT, Pelegrini-Da-Silva A, Parada CA, Fischer L (2012). Effect of gonadal steroid hormones on formalin-induced temporomandibular joint inflammation. Eur J Pain.

[30] Sadamasu A, Sakuma Y, Suzuki M, Orita S, Yamauchi K, Inoue G (2014). Upregulation of NaV1.7 in dorsal root ganglia after intervertebral disc injury in rats. Spine (Phila Pa 1976).

[31] McGowan E, Hoyt SB, Li X, Lyons KA, Abbadie C (2009). A peripherally acting Nav1.7 sodium channel blocker reverses hyperalgesia and allodynia on rat models of inflammatory and neuropathic pain. Anesth Analg.

[32] Lee KS, Zhang Y, Asgar J, Auh QS, Chung MK, Ro JY (2016). Androgen receptor transcriptionally regulates μ-opioid receptor expression in rat trigeminal ganglia. Neuroscience.

